# Psychological abuse is not a problem! Exploring the role of domestic violence myths in psychological revictimization

**DOI:** 10.3389/fpsyg.2023.1228822

**Published:** 2023-10-06

**Authors:** Vincenza Cinquegrana, Maddalena Marini, Silvia Galdi

**Affiliations:** Department of Psychology, University of Campania “Luigi Vanvitelli”, Caserta, Italy

**Keywords:** psychological IPV victimization, IPV myths, acceptance of psychological aggression, revictimization, intimate partner violence

## Abstract

Research provided evidence that IPV myths affect women’s acceptance of psychological aggression in intimate relationships, increasing revictimization. However, no study to date has investigated how endorsement of IPV myths leads victims of psychological IPV to accept psychological aggression. In the present study (*N* = 207 young Italian women involved in heterosexual romantic relationships), we assessed acceptance of IPV myths, prevalence of psychological abuse (in the past 12 months), perception of the problematic nature, and acceptance of psychological aggression in intimate relationships. Results showed that the effect of IPV myths on participants’ acceptance of psychological aggression was mediated by the tendency to consider psychological aggression as unproblematic. Notably, this effect was significant only for women who had experienced some form of psychological abuse by an intimate partner in the past 12 months. These findings have relevant implications for prevention strategies about risks of revictimization.

## Introduction

Psychological abuse in intimate relationships occurs when a person is subjected to actions aimed at preventing or controlling their behavior, causing them emotional harm or fear. These behaviors are characterized in nature by the intent to manipulate, control, isolate, or intimidate the person target of psychological abuse (O’Leary, 1999; Saltzman et al., 2002; Capaldi et al., 2012). Psychological intimate partner violence (psychological IPV) can occur in heterosexual as well as in same-sex intimate relationships (Bartholomew et al., 2008; Walters et al., 2013; Walters and Lippy, 2016; Longares et al., 2018) and it can be perpetrated by men against women, as well as by women against men (Schnurr et al., 2013; Breckenridge et al., 2019). Women are more likely to face psychological abuse at the hands of men they know (Taylor and Mumford, 2016; Fanslow et al., 2023), with one in five having experienced violence at the hands of an intimate partner (European Union Agency for Fundamental Rights, 2014; European Institute for Gender Equality, 2022). As women are disproportionately affected by psychological IPV, the present work focuses on female victims of psychological abuse that occurs in their relationships with men.

Statistics on psychological IPV relate to a cruel reality. For example, according to the EU Agency for Fundamental Rights (FRA), across the EU, 44% of women have experienced psychological IPV in their lifetime (European Union Agency for Fundamental Rights, 2014). Prevalence rates moreover vary greatly across countries (World Health Organization, 2021), suggesting that IPV and the tolerance of abuse by victims are primarily affected by the general culture of a country (Ilies et al., 2003; Meraviglia et al., 2003; McMahon and Banyard, 2012).

The recognition of psychological abuse in intimate relationships as a form of violence is essential, in that it is typically a precursor of other forms of IPV, such as physical and sexual abuse (O’Leary and Smith Slep, 2003; Salis et al., 2014; Cascardi and Avery-Leaf, 2019; Cadely et al., 2020). However, psychological IPV is often underreported or not recognized as it is (Prosman et al., 2014; Myhill, 2017; Brennan et al., 2021). Psychological abuse, indeed, may be quite subtle in nature, covered in pseudo-loving or quasi-humorous tones, and can be exhibited in contexts that minimize its severity, leading victims to less serious perceptions of offenses experienced (Marshall, 1994, 1996, 1999). Marshall (1999), for instance, highlighted how some forms of psychological violence can be subtler than others and difficult to recognize (i.e., manipulation or jealousy); such subtler forms are typically perceived as less coercive and overtly violent than verbal abuse which is readily identifiable as an aggressive act (Marshall, 1999).

In addition, when compared to physical and sexual violence, people tend to consider psychological IPV as less problematic (Capezza and Arriaga, 2008a; Kim and Hogge, 2015; Larsen and Wobschall, 2016; García-Díaz et al., 2017). For example, a recent English study found that police officers are more likely to mention physical assaults and injuries rather than psychological abuse when they are asked to evaluate whether an episode of IPV is “serious” or not (Myhill, 2017). In the same vein, in a qualitative study by Prosman et al. (2014) half of the 14 interviewed abused women did not describe the psychologically abusive behavior of their partner as violent, and, consequently, not worthy of attention from professionals (Prosman et al., 2014).

Overall, this evidence suggests that if we are interested in reducing IPV, efforts should be addressed to investigate psychological abuse, as well as cultural factors that may contribute to placing women at risk for psychological IPV victimization and revictimization (Rivera et al., 2012; Baldry et al., 2016; Keller and Honea, 2016; Baldry and Cinquegrana, 2020; Gutowski and Goodman, 2020; Taccini and Mannarini, 2023). To this end, in the present work, we investigated the normalization of psychologically abusive behaviors as a possible explicative mechanism underlying the relationship between women’s endorsement of IPV myths and acceptance of psychological aggression, and whether such mechanism may help to explain psychological IPV revictimization.

Numerous studies have provided a comprehensive perspective on the issue of IPV, examining various factors across individual, interpersonal, and socio-cultural levels (Capaldi et al., 2012; Costa et al., 2015; Farrington et al., 2017), prioritizing these levels differently in their theories and intervention strategies (Cornelius and Resseguie, 2007; Whitaker et al., 2013; Garzón Segura and Carcedo González, 2020). For instance, clinical psychologists and legal scholars often concentrate on the individual level, delving into the personality traits and characteristics of both IPV perpetrators and victims (Heise, 1998; Testa et al., 2003; Feingold et al., 2008; Debowska et al., 2018; Juarros Basterretxea et al., 2018; Debowska et al., 2019; Baldry and Cinquegrana, 2020). In contrast, feminist sociocultural perspectives, which traditionally emphasize the societal level, view IPV as a significant social manifestation of patriarchy. It is rooted in men’s pursuit of power and the belief that violence is an acceptable means of acquiring and maintaining that power. Feminist scholars, therefore, draw attention to gendered power imbalances within society and prevailing gender ideologies and beliefs (often culturally dominant) as primary sources of IPV (Millet, 1970; Gelsthorpe and Morris, 1990; Messerschmidt, 1993).

According to the feminist perspective, at the societal level, gender ideologies (i.e., Ambivalent Sexism) (Glick et al., 2002), and many beliefs surround IPV. Such beliefs are explored as part of domestic violence myths [i.e., IPV myths; 53–58], a concept that highlights the specific cultural functions of myths. IPV myths can be defined as prejudicial and stereotyped beliefs about violence in intimate relationships (i.e., about its causes, context, consequences, perpetrators, victims, and their interaction) that serve to deny, downplay justify, and legitimate IPV (Lonsway and Fitzgerald, 1995; Faramarzi et al., 2005; Taylor and Sorenson, 2005; Medarić, 2011; Waltermaurer, 2012; Giger et al., 2017; Lelaurain et al., 2018; Yapp and Quayle, 2018; Fakunmoju et al., 2021; Cinquegrana et al., 2022a; Lilley, 2023). These myths encompass notions such as: IPV includes primarily physical and sexual violence by men; a victim of IPV will certainly leave the relationship at once; women who stay with violent partners are complicit in the abuse or are not really being abused; IPV is a relationship issue for which both parties are responsible; and victims of IPV have certain characteristics—for example being provocative (Bograd, 1990; Dobash et al., 1992; Peters, 2003, 2008; Giger et al., 2017; Lelaurain et al., 2018, 2019; Megías et al., 2018).

As the above-mentioned examples highlight, a crucial aspect of IPV myths is that they narrowly define what counts as violence in intimate relationships, suggesting that IPV mainly means “men beating up their wives or girlfriends.” Therefore, IPV myths may help to develop an understanding of IPV that involves physical and/or sexual violence but excludes psychologically abusive situations (Prosman et al., 2014; Cinquegrana et al., 2022a; Toplu-Demirtaş et al., 2022). In addition, IPV myths are linked to a certain reticence to accept IPV as a reality in some sectors of society, justification of the aggression, victim responsibility (for example, previous insults, infidelity, going out without permission, etc.), and exoneration of the perpetrator (Taylor and Sorenson, 2005; European Commission, 2010; Giger et al., 2017; Cinquegrana et al., 2018; Lelaurain et al., 2018, 2019; Fakunmoju et al., 2021), regardless of the gender of the perpetrator (Conroy et al., 2023).

Why do people endorse IPV myths? It has long been posited that IPV myths may serve various psychological functions at both the societal and individual levels. The defensive attribution literature (Walster, 1966; Shaver, 1970a,b; Burger, 1981; Thornton, 1982, 1984; Thornton et al., 1986) posits that IPV myths may help people to understand, explain, and rationalize violence in intimate relationships, maintain cognitive consistency, and fend off negative effects. These defensive attributions can be seen as a manifestation of individuals’ beliefs in a “just world” (Lerner, 1980), according to which good things happen to good people and bad things happen to bad people. By contrast, from a radical feminist perspective, IPV myths may be instrumental in a larger societal function, supporting both patriarchy and violence against women by holding the victim responsible for the abuse, exonerating the perpetrator, and minimizing the seriousness of the crime (Burt, 1980; Adams, 1988; Bograd, 1990; Dobash et al., 1992; Giger et al., 2017), thus reducing social support for victims (Waltermaurer, 2012; Fleming and Franklin, 2021; Pagliaro et al., 2021; Wijaya et al., 2022). According to this perspective, therefore, IPV is a manifestation of gender inequality and a mechanism for the control and subordination of women by men (Burt, 1980).

IPV myths, moreover, have divergent functions for the two genders. These myths allow men to rationalize and justify abusive behaviors, as well as avoid anticipated blame (Lerner and Matthews, 1967; Finke, 1995), by trivializing violence in intimate relationships, blaming the victim, and justifying the perpetrator (Tang et al., 2002; Medarić, 2011; Waltermaurer, 2012; Giger et al., 2017; Lelaurain et al., 2018; Fakunmoju et al., 2021). Conversely, IPV myths allow women to minimize personal vulnerability; to uphold a perception of safety, some women, indeed, may rely on IPV myths to distance themselves from the reality of the violence (Thornton, 1984; Thornton et al., 1986). Women who embrace IPV myths tend to align with inflexible gender roles and subscribe to ambivalent sexism, wherein women who challenge traditional roles face consequences (hostile sexism) and those who follow them will be protected (benevolent sexism) (Glick et al., 2002; Allen et al., 2009; Brandt, 2011; Alvarez et al., 2021; Cinquegrana et al., 2022a), believing that violence in intimate relationships mainly happens to a certain type of women [e.g., career women (Capezza and Arriaga, 2008b); women who exhibit provocative behaviors (Esqueda and Harrison, 2005); verbally aggressive women (Witte et al., 2006)], thus protecting themselves from the psychological threat of being a potential victim. Women who reject IPV myths, by contrast, believe that any woman can be a victim of IPV and, therefore, perceive IPV as a potential threat to all women, including themselves. In sum, IPV myths serve as an anxiety buffer to women who accept them: the more women endorse IPV myths, the less threatened and vulnerable they feel about their possibility of victimization.

Overall, this evidence shows that acceptance of IPV myths among both (potential) perpetrators and victims may be a powerful tool for perpetrating IPV. More importantly, it suggests that to the extent they endorse IPV myths, the likelihood increases for women to justify abusive behaviors in intimate relationships (Medarić, 2011; Giger et al., 2017; Lelaurain et al., 2018; Megías et al., 2018). As a result, women will be more likely to accept this form of violence perpetrated against them, thus increasing the risks of victimization (Faramarzi et al., 2005; Rodríguez-Franco et al., 2012; Mugoya et al., 2015; Spencer et al., 2019; Nazar et al., 2021; Cinquegrana et al., 2022a; Kadengye et al., 2023). Consistently, a recent study by Cinquegrana et al. (2022a) has shown that greater endorsement of IPV myths predicted a higher prevalence of psychological IPV victimization among women through the mediating role of acceptance of psychological aggression. These findings are especially instructive because they clearly demonstrate that acceptance of IPV myths increases women’s acceptance of psychological aggression, thus fostering psychological IPV revictimization. This is critical since psychological violence is typically the first to appear in an abusive relationship and often a means to prepare the ground for other abusive or violent behaviors (O’Leary and Smith Slep, 2003; Luzón et al., 2011; Salis et al., 2014; Cascardi and Avery-Leaf, 2019; Cadely et al., 2020).

Why do victims who endorse IPV myths often accept the experience rather than report the incident to authorities? Such reactions may have different explanations. Endorsement of IPV myths may indeed lead victims, among others, to fear lack of support, fear of being blamed for the incident or of not being believed, fear that the occurrence was not harmful enough, and/or to attribute blame for the incident to themselves, thus justifying the perpetrator (Medarić, 2011; Waltermaurer, 2012; Giger et al., 2017; Lelaurain et al., 2018; Megías et al., 2018). In the present work, we proposed and tested an additional reason. As discussed above, IPV myths contribute to a public misunderstanding of what constitutes abuse in intimate relationships, mainly confining violence in intimate relationships to physical and sexual abuse. This disconnection between individuals’ ideas about IPV and the reality of most actual psychological IPV experiences may help to explain why so many victims themselves do not conceptualize their experience of psychological IPV as abuse, with these victims instead misperceiving personal experience as a more normal event. It seems, therefore, plausible that acceptance of psychological abuse would be explained by victims’ downplaying of the problematic nature of psychologically aggressive behaviors.

To the best of our knowledge, no study to date has investigated whether (a) the (mis) perception of psychological abuse can help to explain the relationship between endorsement of IPV myths and acceptance of psychological abuse, and (b) the role played by (mis) perception of psychological abuse in the relationships between IPV myths and acceptance of psychological abuse differ for victims and no victims of psychological abuse. Investigating whether women’s (mis)perception of psychological abuse plays a role in the relationship between endorsement of legitimatizing myths of IPV and acceptance of psychological abuse is crucial not only for improving our understanding of women’s IPV victimization but also for implementing interventions aimed at reducing the cumulative harm of the violence in intimate relationships and risks of revictimization.

## Overview of the current study

**Figure 1:**
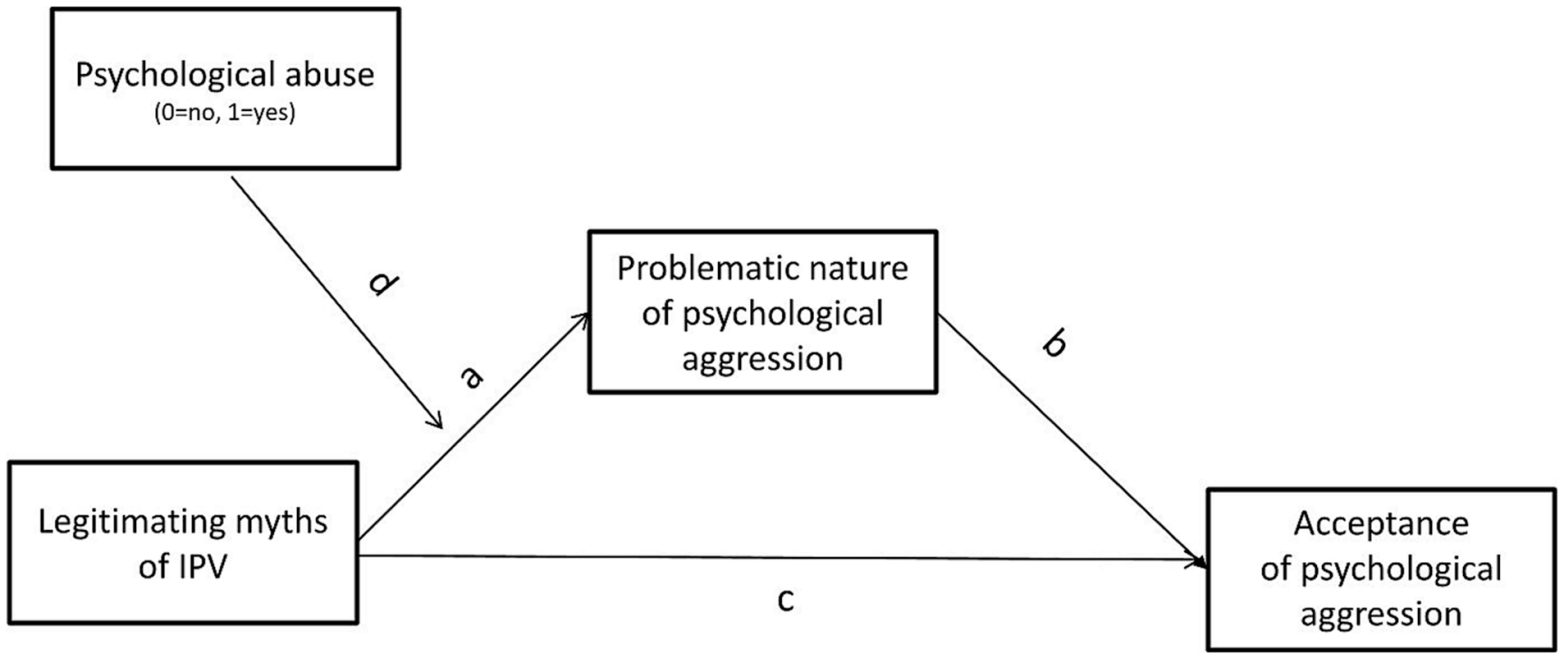
Proposed research model.

IPV myths play a crucial role in the acceptance of psychological IPV. Moreover, past histories of IPV victimization may be related to the abuse perception since the more women manifest acceptance of IPV, the more vulnerable they are to experience it (Faramarzi et al., 2005; Rodríguez-Franco et al., 2012; Mugoya et al., 2015; Spencer et al., 2019; Nazar et al., 2021; Cinquegrana et al., 2022a; Kadengye et al., 2023). Based on this evidence, our working model was to analyze whether the normalization of psychological aggression, namely the perception of psychological aggression as unproblematic, may account for the relationship between women’s acceptance of IPV myths and acceptance of psychological aggression in intimate relationships, taking into account the objective existence, or not, of past experiences of psychological abuse (see Figure 1).

We expected that the effect of endorsement of IPV myths on the acceptance of psychological abuse would be mediated by the perception of psychological aggression in intimate relationships as unproblematic and moderated by past experiences of psychological abuse.

## Method

### Participants

Female students of an introductory course in Psychology were invited to participate in an online survey to investigate their beliefs and opinions on romantic relationships. The convenience sample comprised a total of 207 young women who volunteered for the study. Participants were all heterosexual and aged between 18 and 26 (*M*age = 20.26; SD = 1.52); 23 (11.1%) were residents of Northern Italy, 85 (41.1%) residents of Central Italy, and 99 (47.8%) residents of Southern Italy. All participants had a high school degree and were in a romantic relationship (*M_Months_* = 27.63; *SD* = 24.63). All respondents provided their informed consent to participate in the study, in accordance with the Declaration of Helsinki.

The procedure and materials of the study were approved by the University Ethics Committee for Psychological Research. This study was carried out in accordance with the recommendations of the following Italian (Law 101 of 2018) and European (Law 679 of 2016- General Data Protection Regulation) laws which regulate the Code of Conduct for Italian psychologists about standards for research activities.

### Procedure

The survey was created using the software SurveyMonkey and designed to avoid any missing data.

After reading the consent form and accepting to participate in the study, participants filled out a questionnaire. Participants provided their socio-demographic information and completed a scale aimed at assessing acceptance of IPV myths (i.e., the Domestic Violence Myth Acceptance Scale — DVMAS) (Peters, 2008) and a measure to determine whether they had experienced some form of psychological abuse by an intimate partner during the last 12 months (i.e., the Measure of Psychologically Abusive Behaviors, MPAB) (Follingstad et al., 2015). Then, participants were required to rate 14 brief scenarios describing the real-life situations of a young couple. In each scenario, the woman was confronted with psychologically aggressive behavior by her partner. Participants were instructed to place themselves in the female protagonist role and indicate how acceptable and problematic they considered each behavior of psychological aggression. At the end of the survey, participants were thanked for their participation, fully debriefed, and asked to confirm or reject their consent to data processing. All participants provided consent.

In accordance with the ethical guidelines outlined by the World Health Organization in 2001 (World Health Organization, 2001) for conducting population-based surveys on IPV against women, our approach involved querying respondents about their personal experiences with specific acts, rather than employing terms such as “abuse” or “violence.” Furthermore, upon the completion of the study, we extended an invitation to participants for a feedback session. During this session, participants received the study’s findings, information related to IPV and gender-related issues, as well as details about the available health, legal, social services, and educational resources within the community. This comprehensive approach aimed to identify the various forms of support each resource could offer, regardless of whether participants had disclosed or recognized their experiences of violence.

### Materials and measures

#### Demographics

Participants responded to the following socio-demographic questions: age, gender, sexual orientation, level of education, and relationship status.

#### Acceptance of IPV myths

Participants’ acceptance of IPV myths was assessed using the Domestic Violence Myth Acceptance Scale (DVMAS) (Peters, 2008). The DVMAS is an 18-item self-report questionnaire developed to evaluate cultural beliefs that serve to legitimize and perpetuate violence in IPV. It includes four dimensions: character blame (e.g., “If a woman stays with the man who abused her, she basically deserves what she gets”), behavior blame (e.g., “Domestic violence occurs because women keep on arguing about things with their partners”), exoneration of the perpetrator (e.g., “Domestic violence results from a momentary loss of temper”), and minimization of the violence (e.g., “Domestic violence does not affect many people”). The overall scale has shown internal consistency across different socio-cultural contexts (Peters, 2008; Giger et al., 2017; Cinquegrana et al., 2022a). Participants rated each item on a 7-point scale ranging from 1 (*strongly disagree*) to 7 (*strongly agree*). In this study, the internal consistency of the DVMAS was satisfactory (Cronbach’s *α* = 0.70) and comparable to that of previous studies with Italian participants (Cinquegrana et al., 2022a). Scores for acceptance of IPV myths were computed by averaging participants’ responses to the 18 items, such that higher scores reflected a greater endorsement of these false beliefs.

#### Prevalence of psychological abuse

To assess participants’ psychological IPV during the last 12 months, we employed the Measure of Psychologically Abusive Behaviors (MPAB) (Follingstad et al., 2015). The MPAB is commonly used to identify behaviors for which recipients believe their partner intentionally acted psychological harm. The MPAB includes 14 categories of psychological aggression. Each category consists of three items representing increasingly severe actions (mild, moderate, and severe), with mild actions being only relatively less abusive than moderate or severe level items. For this study, we selected the 7 most frequent categories of psychological abuse, which are also sneakier in their mild, moderate, and severe actions (Follingstad et al., 1990; Harned, 2001; Carney and Barner, 2012; Follingstad et al., 2015).

Therefore, in the present abridged form, the MPAB contained 21 items measuring: monitoring (3 items; e.g., “He tried to make you report on details about where you went and what you did when you were not with him, as a way to check on you”), jealousy (3 items; e.g., “He acted very upset because he felt jealous when you spoke to, or looked at, another man, so that you had to restrict your behavior toward others”), verbal abuse (3 items; e.g., “He criticized and belittled you as a way to make you feel bad about yourself”), isolating (3 items; e.g., “He acted rude toward, gossip about, or tell lies about your family and friends, to discourage you from spending time with them”), public humiliation (3 items; e.g., “He threatened to reveal an embarrassing secret, as a way to hurt or manipulate you”), manipulation (3 items; e.g., “He continued to act very upset – e.g., pouted, stayed angry, gave you a silent treatment – until you did what he wanted you to do”), and creating a hostile environment (3 items; e.g., “He intentionally turned a neutral interaction into an argument or disagreed with you with the purpose to create conflict”). For each item, participants indicated how often they had experienced that abusive behavior within the last 12 months on a scale ranging from 0 (*never*) to 5 (*almost daily*). In line with Follingstad et al. (2015), each item was coded as 1 when participants reported having experienced the abusive behavior during the last 12 months (regardless of its frequency) and 0 when they indicated to had not. Then, for each participant scores on the 21 items were summed. Therefore, the index of the prevalence of psychological abuse could range from 0 (i.e., never experienced psychological abuse in the last 12 months) to 21 and reflects the number of psychologically abusive behaviors included in the MPAB that participants had experienced in the last year.

#### Problematic nature of psychological aggression and acceptance

We used short hypothetical scenarios (for a similar procedure Herzog, 2007; Yamawaki et al., 2009; DeHart et al., 2010; Nguyen et al., 2013; Cinquegrana et al., 2022a,b) to assess the extent to which participants perceived behaviors of psychological aggression in intimate relationships as problematic and the acceptability of psychological aggression in intimate relationships. To this end, we selected the same 7 categories of psychological abuse included in our brief version of the MPAB (i.e., monitoring, jealousy, verbal abuse, isolating, public humiliation, manipulation, and creating a hostile environment). We then constructed 14 scenarios describing the daily life episodes of a young woman and her partner (named “S.”). Each scenario referred to a specific category of psychological abuse and incorporated a specific example of action fitting within the mild or moderate level of the egregiousness of the MPAB. For instance, a scenario dealing with a mild action of jealousy read: “A guy has looked at you and S. has noticed the event. He gets mad at you and accuses you of having looked at that guy intentionally” (for more details see Cinquegrana et al., 2022a). Since we were interested in the subjective view of participants, unlike the MPAB, the likely malignant intention of the perpetrator was excluded from all descriptions. For each scenario, participants were instructed to place themselves in the role of the female protagonist and to indicate how problematic and acceptable they considered the behavior enacted by the woman’s partner, on two scales ranging from 1 (*not at all*) to 10 (*entirely*). Mean scores of the perceived problematic nature of psychological aggression (*α* = 0.81) and acceptability of psychological aggression (*α* = 0.74) were then calculated. Higher scores of the two indices indicated a greater perception of psychological aggression as unproblematic and a greater acceptance of psychological aggression.

#### Plausibility check

Participants estimated how real the actions described in the scenarios were on a scale ranging from 1 (*not at all*) to 7 (*entirely*). Actions were judged by participants as real (*M* = 6.24; *SD* = 1.02; range 2–7), suggesting that a fairly good job was done to construct the scenarios.

## Results

### Descriptive statistics and correlations

Descriptive statistics and correlations among study variables (i.e., acceptance of IPV myths, perceived problematic nature of psychological aggression, acceptance of psychological aggression, and prevalence of psychological abuse) are presented in Table 1. Overall, participants showed low levels of acceptance of IPV myths (*M* = 2.12; *SD* = 0.55). Moreover, respondents judged as acceptable almost no one of the psychologically aggressive behaviors described in the scenarios (*M* = 1.67; *SD* = 0.88) and perceived almost all of them as problematic (*M* = 8.85; *SD* = 1.22). As expected, acceptance of IPV myths correlated positively with scores of acceptability of psychological aggression, while negative correlations emerged between the acceptance of IPV myths and the perceived problematic nature of psychological aggression.

**Table 1:** Means, standard deviations, and zero-order correlations among study variables (acceptance of IPV myths, perceived problematic nature of psychological aggression, acceptance of psychological aggression, and prevalence of psychological abuse).

Variables	Correlations				
	*M* (*SD*)	1	2	3	4
1. Acceptance of IPV myths	2.12 (0.55)	–			
2. Perceived problematic nature of psychological aggression	8.85 (1.22)	−0.32^**^	–		
3. Acceptance of psychological aggression	1.67 (0.88)	0.31^**^	−0.59^**^	–	
4. Prevalence of psychological abuse	4.09 (4.82)	0.03	−0.21^**^	0.13	–

*N* = 207. **p < 0.01.

As shown in Table 2, responses to the MPAB revealed that only 49 participants (23.7%) had never experienced psychological abuse during the last 12 months, whereas 158 (76.3%) had experienced at least one of the 21 abusive behaviors. Among the subtler forms of psychological abuse (Marshall, 1999), the most frequently reported categories were the creation of a hostile environment (*n* = 126), manipulation (*n* = 92), and jealousy-driven restrictions (*n* = 76). These were followed by a more overt form, verbal abuse (*n* = 80). We then classified participants into two groups: victims of psychological abuse (i.e., victim, coded as 1), when one or more of the acts described on the MPAB occurred in the past year, and non-victims of psychological abuse (i.e., non-victim, coded as 0), when no acts occurred in the last year. Abused and no abused women did not differ significantly either in the acceptance of IPV myths (*t* = −0.99_(205),_ *p* = 0.33) or in acceptance of psychological aggression [*t* = −1.84_(205),_ *p* = 0.07].

**Table 2:** Prevalence of psychological abuse as a function of the 7 categories included in the measure of psychologically abusive behaviors (MPAB).

Categories	No		Yes	
	*N*	%	*N*	%
Isolate	159	76, 8	48	23, 2
Manipulate	115	55, 6	92	44,4
Public humiliation	168	81, 2	39	18, 8
Verbal abuse	127	61, 4	80	38, 6
Hostile environment	81	39, 1	126	60, 9
Monitor	161	77, 8	46	22,3
Restriction due to Jealousy	131	63, 3	76	36, 7

*N* = 207.

### Moderated mediation model

**Figure 2:**
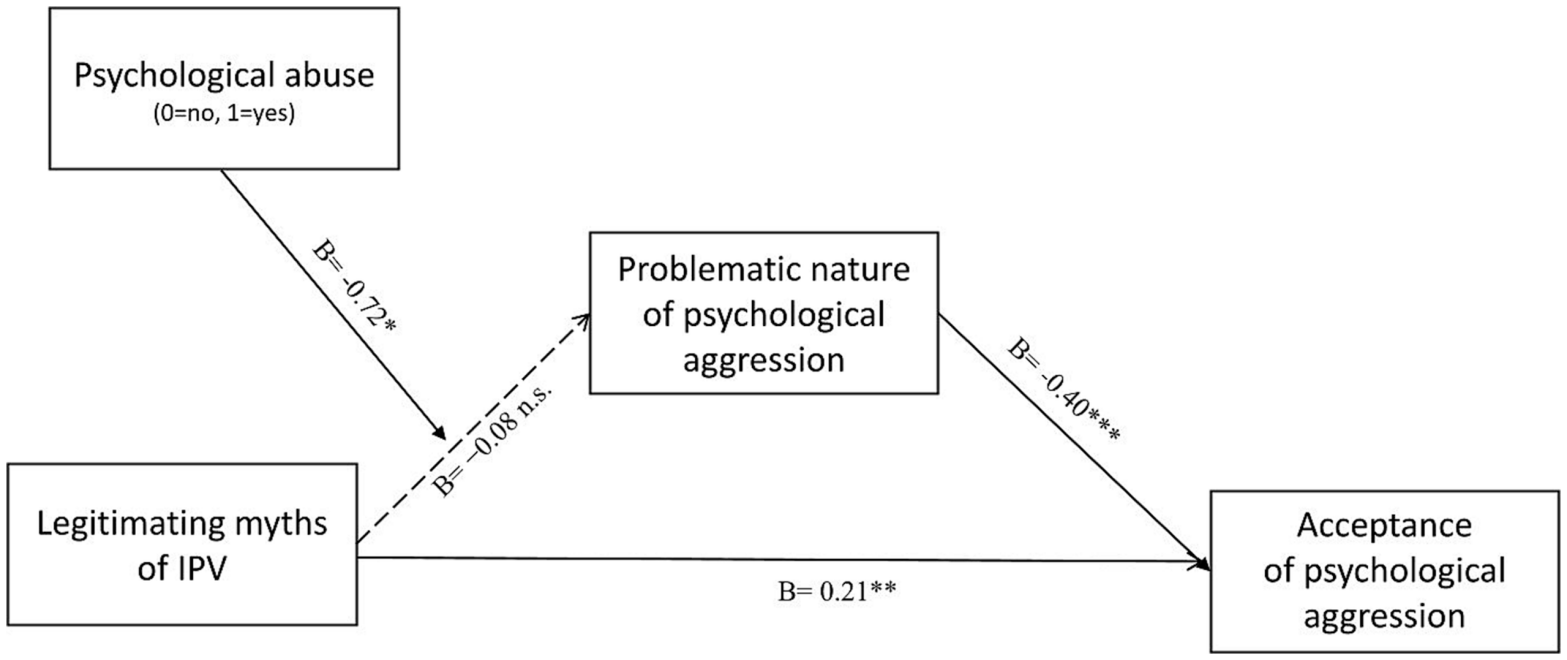
Results of the moderated mediation model.

**Figure 3:**
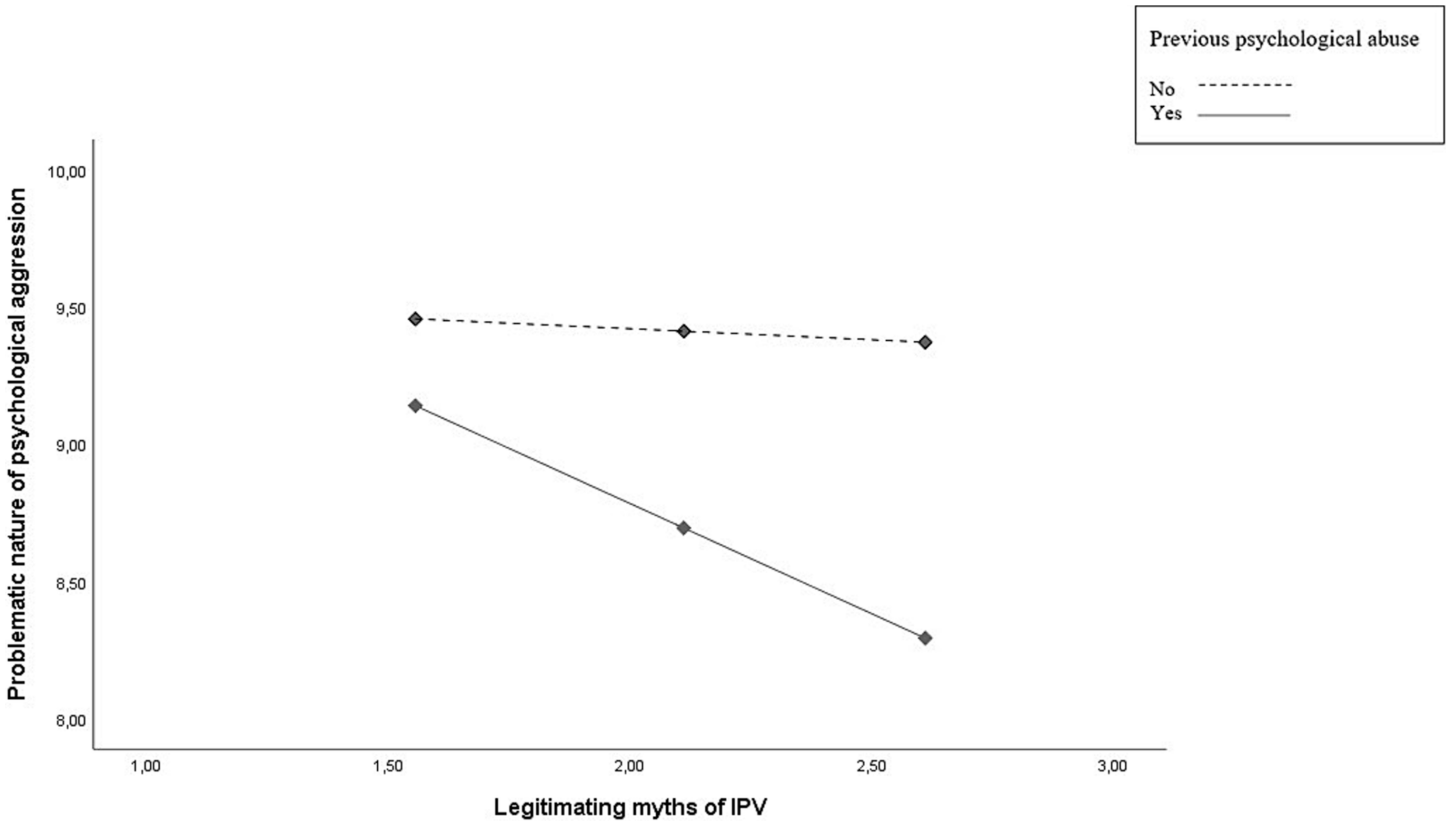
Simple slope analysis.

To test our hypothesis, we performed a moderated mediation model on the PROCESS macro for SPSS (Model 7) (Hayes, 2022), with 5,000 bootstrap resamples and 95% confidence intervals. In the model, acceptance of IPV myths was the independent variable (X), psychological abuse was the moderator (W; no-victim = 0, victim = 1), the perceived problematic nature of psychological aggression was the mediator (M), and acceptance of psychological aggression was the dependent variable (Y). The overall moderated mediation model attained statistical significance, *Index* = 0.29, *SE* = 0.13, 95% CI [0.05, 0.58]. Results are illustrated in Figure 2. No main effect of acceptance of IPV myths, *B* = −0.08, *SE* = 0.32, *t* = −0.25, *p* = 0.80; 95% CI [−0.71, 0.55] and psychological abuse, *B* = 0.81, *SE* = 0.76, *t* = 1.06, *p* = 0.29; 95% CI [−0.69, 2.30] on perceived problematic nature of psychological aggression was found. Moreover, acceptance of IPV myths had a significant main positive effect on acceptance of psychological aggression, *B* = 0.21, *SE* = 0.09, *t* = 2.28, *p* = 0.02; 95% CI [0.03, 0.39], and perception of the problematic nature of psychological aggression had a significant main negative effect on acceptance of psychological aggression, *B* = −0.40, *SE* = 0.04, *t* = −9.34, *p* < 0.001; 95% CI [−0.48, −0.31]. Importantly, as expected, the two-way interaction between acceptance of IPV myths and psychological abuse on the perceived problematic nature of psychological aggression was significant, *B* = −0.72, *SE* = 0.36, *t* = −2.02, *p* = 0.04; 95% CI [−1.42, −0.02]. The simple slopes analysis (see Figure 3) revealed that acceptance of IPV myths reduced the perceived problematic nature of psychological aggression when women had experienced psychological abuse during the last 12 months, *B* = −0.80, *SE* = 0.15, *t* = −5.17, *p* < 0.001; 95% CI [−1.11, −0.50], whereas no significant relation was found between acceptance of IPV myths and perceived problematic nature of psychological aggression for women who had not experienced psychological abuse during the last 12 months, *B* = −0.08, *SE* = 0.32, *t* = −0.25, *p* = 0.80; 95% CI [−0.71, 0.55]. Moderated mediation analysis revealed that the perceived problematic nature of psychological aggression was a significant mediator for women with past experiences of psychological abuse, *B* = −0.32, *SE* = 0.10; 95% CI [0.14, 0.54], but not for women who had not such experiences, *B* = 0.03, *SE* = 0.09; 95% CI [−0.15, 0.23].

## Discussion

Psychological IPV is the most frequent form of gender-based violence, especially among young women (European Union Agency for Fundamental Rights, 2014; Taylor and Mumford, 2016; SafeLives, 2019; European Institute for Gender Equality, 2022). Although it has been recognized as a precursor of physical and sexual violence (O’Leary and Smith Slep, 2003; Salis et al., 2014; Cascardi and Avery-Leaf, 2019; Cadely et al., 2020), considered the most extreme and visible form of IPV, psychological abuse is often undervalued by victims, as well as professionals (Prosman et al., 2014; Myhill, 2017; Heise et al., 2019; Brennan et al., 2021; World Health Organization, 2021).

According to feminist approaches and international organizations (Bograd, 1990; Dobash et al., 1992; European Commission, 2010; World Health Organization, 2012, 2016, 2021), an important risk factor in the ability to recognize psychological IPV as a form of violence is represented by a set of false beliefs, defined as domestic violence myths (i.e., IPV myths; Bograd, 1990; Dobash et al., 1992; Peters, 2008; Giger et al., 2017; Lelaurain et al., 2018, 2019), which contribute toward the pervasiveness of abuse in intimate relationships by holding the victim responsible for the abuse, exonerating the perpetrator, and minimizing the seriousness of the crime (Kilpatrick, 2004; Peters, 2008; Giger et al., 2017; Cinquegrana et al., 2018; Lelaurain et al., 2018, 2019). Research has shown that endorsement of IPV myths fosters women’s acceptance of psychological abuse (Medarić, 2011; Megías et al., 2018; Cinquegrana et al., 2022a) and, consequently, the prevalence of psychological IPV victimization (Rodríguez-Franco et al., 2012; Mugoya et al., 2015; Spencer et al., 2019; Nazar et al., 2021; Cinquegrana et al., 2022a; Kadengye et al., 2023). Drawing from this evidence, in the present work we examined the mechanism through which myths of IPV may lead to the acceptance of psychological violence in heterosexual intimate relationships. Because IPV myths mainly confine IPV to physical and sexual abuse, we hypothesized that these beliefs would increase the normalization of psychological violence, that is the perception of psychological aggression as unproblematic. More importantly, we investigated whether this mechanism plays a role in revictimization and, therefore, differs in victims and no-victims of psychological abuse.

In line with previous studies (Stanley, 2012; Giger et al., 2017), our results showed that victims and no-victims did not differ in their levels of acceptance of IPV myths. Moreover, confirming past evidence, we found that greater endorsement of these beliefs predicted greater acceptance of psychological aggression in intimate relationships (Faramarzi et al., 2005; Medarić, 2011; Megías et al., 2018; Cinquegrana et al., 2022a). More importantly, results revealed that for victims, but not for no-victims, the relationship between acceptance of IPV myths and acceptance of psychological aggression was explained by the misperception of psychological aggression as unproblematic. Therefore, the findings of the present study offer a clear contribution to the literature by providing evidence that victims may not perceive (at least some) psychologically aggressive behaviors as such (Harris et al., 2005; Prosman et al., 2014), but consider them as unproblematic, thus accepting them.

We are hopeful that findings from the present study might be helpful in planning prevention and intervention strategies and policies aimed at curbing IPV victimization. Given that IPV typically begins with fairly subtle controlling and coercive behaviors which typically escalate over time (Winstok, 2013; Natarajan, 2017), helping women to promptly recognize violent and abusive psychological behaviors is an important step in establishing healthy and safe relationships and can also increase their awareness of resources available to them if they are in a violent relationship.

Intervention programs should be grounded in the understanding that IPV is a complex phenomenon influenced by multiple risk factors across different levels (Costa et al., 2015; Farrington et al., 2017; Garzón Segura and Carcedo González, 2020). This includes societal risk factors that permeate the environments in which victims reside, often normalizing abusive behaviors and making it challenging to recognize what constitutes violence.

Specifically, intervention programs should focus on (i) sharing information about definitions, signs, and consequences of psychological IPV, and (ii) explaining the role of acceptance of IPV myths, emphasizing that they contribute to the misperception of what is psychological violence, thus representing a dangerous factor that triggers revictimization.

There are some potential limitations. In this study, we provide only cross-sectional data, which does not allow attesting to any causal links. So, future studies should replicate these findings by employing a longitudinal design and examining the change in psychological IPV victimization over time. Another limitation of this study is its exclusive focus on a single risk factor related to psychological IPV. Future research endeavors should broaden their scope to encompass a multitude of sociocultural factors by incorporating various risk elements at different levels of analysis. Furthermore, future studies should include an examination of romantic beliefs. These beliefs, centered around the idea of eternal love conquering all obstacles, bear a resemblance to the myths explored in this study and could potentially contribute to the persistence of unhealthy relationships (Papp et al., 2017; Carbonell Marqués and Mestre, 2019; Ferrer Pérez et al., 2019; Fernández et al., 2023).

Despite these limitations, the present work contributes to our understanding of the possible mechanism of women’s psychological IPV revictimization, in order to implement more effective intervention and prevention strategies.

## Data availability statement

The raw data supporting the conclusions of this article will be made available by the authors, without undue reservation.

## Ethics statement

The studies involving humans were approved by Ethics Committee of the Department of Psychology, University of Campania “Luigi Vanvitelli.” The studies were conducted in accordance with the local legislation and institutional requirements. The participants provided their written informed consent to participate in this study.

## Author contributions

All authors listed have made a substantial, direct, and intellectual contribution to the work and approved it for publication.
